# *TWEAK/Fn14* hypomethylation and higher plasma TWEAK and TNF-α levels are related to sarcopenic obesity in community-dwelling elderly in Xinjiang

**DOI:** 10.1097/MD.0000000000042937

**Published:** 2025-07-04

**Authors:** Lingling Ma, Zhuoya Maimaitiwusiman, Saiyare Xuekelati, Buluhan Halan, Jinling Liu, Hongmei Wang

**Affiliations:** a Xinjiang Medical University, Urumqi, Xinjiang, China; b Second Department of Comprehensive Internal Medicine of People's Hospital of Xinjiang Uygur Autonomous Region, Urumqi, Xinjiang, China.

**Keywords:** community-dwelling elderly, methylation, sarcopenic obesity, TNF-α, TWEAK/Fn14

## Abstract

Sarcopenic obesity is a primary factor contributing to the decline in the quality of life among the elderly in our country. Studies have shown that hypomethylation of tumour necrosis factor-like weak inducer of apoptosis (TWEAK)/fibroblast growth factor-inducible 14 (Fn14) is associated with sarcopenia. Our research primarily explores whether this mechanism also affects the progression of sarcopenic obesity. A total of 120 subjects were grouped into the case (sarcopenic obesity) and control (non-sarcopenic and nonobese) groups, with 60 subjects in each group based on power test (medium effect size, power = 80%). Bisulfite sequencing was used to assess the methylation status of TWEAK/Fn14 in these subjects. ELISA was employed to measure the plasma concentrations of TWEAK, tumour necrosis factor-alpha (TNF-α), interleukin (IL)-4, IL-6, and IL-10. SPSS 17.0 and R software was used for statistical analysis, employing multiple logistic regression to adjust for residual confounding factors, and Student *t* tests for difference analysis. Compared to the case group, the control group had higher methylation levels at CpG20, CpG30 sites in TWEAK (*P* = .043 for both). Additionally, the control group showed higher methylation level at CpG25 site in Fn14 (*P* = .025). After adjusting for hypertension, diabetes, triglyceride levels, gender, and age, Student *t* tests covariance confirmed significant differences in the enrichment levels of TWEAK, TNF-α, IL-10, and IL-4 of patients in case group (*P*_TWEAK_ = .017, *P*_TNF-α_ < .001, *P*_IL-10_ = .010, *P*_IL-4_ = .016). Multivariate logistic regression analysis revealed that higher plasma levels of TWEAK and TNF-α were significantly associated with the incidence of sarcopenic obesity (odds ratios = 1.024, 95% confidence interval = 1.006–1.042; odds ratios = 2.537, 95% confidence interval = 1.697–3.793). This evidence suggested that hypomethylation of TWEAK/Fn14 also affected sarcopenic obesity progression. Increased enrichment of TWEAK and TNF-α in plasma, along with hypomethylation of TWEAK and Fn14, promotes the occurrence of sarcopenic obesity may affect occurrence of sarcopenic obesity.

## 1. Introduction

With the progressive aging of the global population, age-related health issues have emerged as an increasingly pressing cause of public health concern. Sarcopenic obesity is a recently defined geriatric syndrome characterized by the concurrent presence of obesity and sarcopenia, leading to an age-related decrease in skeletal muscle mass, strength, and function.^[1–3]^ Over the next 35 years, sarcopenic obesity is forecast to impact 100 to 200 million people throughout the world.^[4]^ This syndrome may lead to an increase in cardiovascular and metabolic diseases, as well as an increase in disability and mortality rates. This suggests that sarcopenia and obesity may independently or synergistically contribute to negative patient outcomes.^[5,6]^ The dual metabolic pressures imposed by obesity and sarcopenia can exacerbate the risk of frailty, falls, hospitalization, disability, and mortality while also contributing to a worse quality of life for elderly individuals.^[7–9]^ Sarcopenic obesity thus imposes an immense burden on affected individuals, their families, public healthcare, and society as a whole.

The specific pathogenic factors that contribute to the incidence of sarcopenic obesity remain poorly understood, and studies that offer insight into the factors contributing to this condition have the potential to guide preventative or therapeutic interventions. DNA methylation is a common epigenetic modification form. Methyltransferases selectively methylate cytosine residues within CpG dinucleotides (cytosine followed by guanine), particularly in regions rich in CpG sites known as CpG islands, generating 5-methylcytosine,^[10]^ this leads to changes in chromatin structure, DNA conformation, DNA stability, and DNA–protein interactions, thereby altering the expression of specific genes. There are a large number of differential methylation sites in blood samples from obese patients,^[11]^ and specific CpG loci of methylation is associated with obesity.^[12]^ Prior research has revealed an important role for altered DNA methylation affecting obesity progression.^[13–19]^

Many different factors are believed to contribute to the incidence of sarcopenic obesity including insulin resistance, aging-related changes in hormone production, altered energy metabolism, and chronic low-grade inflammation.^[20,21]^ Inflammatory activity is also a hallmark of obesity, which is characterized by higher proinflammatory cytokine expression together with reduced anti-inflammatory cytokine levels in adipose tissue and extensive macrophage infiltration. Tumor necrosis factor-like weak inducer of apoptosis (TWEAK) and its receptor fibroblast growth factor-inducible 14 (Fn14) have both been shown to play important functional roles in the pathogenesis of insulin resistance, obesity, and other metabolic disorders.^[21]^ Our previous research indicates that the hypomethylation of TWEAK and Fn14 are promoted the incidence of sarcopenia.^[22]^ Other studies have also confirmed that tumour necrosis factor-alpha (TNF-α) and TWEAK levels in plasma are abnormally enriched in sarcopenic and obese patients.^[23]^ Combining previous data, we speculate that these changes might occur in patients with sarcopenic obesity.

Therefore, this study aims to investigate the underlying association of DNA methylation characteristics in the elderly population with sarcopenic obesity in the Xinjiang region. Firstly, we provide evidence that there is an abnormal methylation status of TWEAK and Fn14 in the plasma of patients with sarcopenic obesity. The inflammatory factor TNF-α is associated with this process. Our study contributes to the identification of promising therapeutic targets for the prevention or treatment of this disease.

## 2. Methods

### 2.1. Study population

The Ethics Committee of the People’s Hospital of Xinjiang Uygur Autonomous Region (Xinjiang, China) approved this study (Ethical numbers: For National Science Foundation of China 2016011). All participants provided written informed consent for study participation. We conducted the patient selection as previously described in our research.^[23]^ Briefly, a typical group of elderly individuals living in communities across Xinjiang was gathered using a 3-tiered stratified sampling method. During the initial phase, a county from both the northern and southern regions of Xinjiang was picked randomly (specifically, Mulei County in the north and Luopu County in the south). In the subsequent phase, 6 townships within each county were chosen randomly, followed by the random selection of 5 villages within each township in the final phase. To ensure the representative nature of population samples, the results of the sixth national Chinese census were used for sample weighting.

*Inclusion criteria:* community-dwelling elderly aged 60 and above, regardless of gender, who are permanent residents of the Xinjiang Uyghur Autonomous Region.

*Exclusion criteria:* this study excluded participants who exhibited the following conditions: (1) mental illness; (2) severe cognitive impairment; (3) vital organ failure history; (4) history of surgery, recent infection, or acute cardiovascular disease; (5) stroke sequelae; (6) consumptive diseases including tuberculosis and malignant tumors.

The epidemiological survey ultimately included 2100 cases, of whom 1876 completed the survey (response rate: 89.33%).

*Physical examination:* under the guidance of clinicians, participants completed a set of standardized questionnaires.^[23]^ Standard methods were employed by trained staff to collect patient parameters including weight, height, blood pressure, and waist circumference.

*Skeletal muscle mass index:* The Biospace Inbody 720 (Seoul, South Korea) is used for detecting bioelectrical impedance analysis to estimate skeletal muscle mass. The calculation method for skeletal muscle mass index is to divide the skeletal muscle mass (kg) by the square of the body height (m^2^).

*Physical fitness assessment: w*e used the typical gait speed within a 6-meter distance to evaluate physical performance, and calculated walking speed from the time taken to complete the Timed Up and Go (TUG) test. The formula is: walking speed = [6/(TUG_completion time_)]×1.62, to convert the TUG completion time into a standardized walking speed measurement.

*Dominant hand grip strength:* JAMAR hand-held dynamometers (Sammons, USA) were used to measure dominant hand grip strength. The participants stand with their feet apart, shoulder-width apart. A hand-held dynamometer is used to measure grip strength. Participants are instructed to remain still and exert their maximum force, with each hand tested once, recording the maximum grip strength of the participants, accurate to 0.1 kg.

*Blood plasma sample collection:* blood samples were collected from study participants after overnight fasting (from 8 pm to 8 am) and assessed for serum biochemical parameters as well as the methylation status of *TWEAK* and *Fn14*. After collection, these samples were collected by county hospitals prior to shipping by air to Urumqi where they were stored at −80 °C prior to laboratory assays that were conducted in the Clinical Laboratory Center of the People’s Hospital of Xinjiang Uygur Autonomous Region (Grade 3A Hospital).

### 2.2. Grouping of study participants

In total, 120 individuals were selected at random from a database based upon the results of the epidemiological survey detailed above (setting medium effect size (d = 0.5) with α = 0.05 and Power = 80% (β = 0.2) in power analysis) through computer-generated balanced block randomization, randomization into 2 groups, including 60 subjects with sarcopenic obesity and 60 non-sarcopenic, nonobese subjects. For further details regarding this selection process, see our prior study.^[23]^

### 2.3. Diagnostic criteria

*Sarcopenia:* defined in accordance with the Asian Working Group for Sarcopenia 2014^[24]^ consensus diagnostic criteria. Specifically, participants were diagnosed with sarcopenia if they exhibited a reduced appendicular skeletal muscle index (females < 5.7 or males < 7.0 kg/m^2^) together with reduced handgrip strength (females < 18 or males < 26 kg) or < 0.8 m/s reduced gait speed.

*Obesity:* obesity was defined by a waist circumference ≥ 85 and ≥ 80 cm for males and females, respectively, in accordance with the guidelines for the prevention and control of overweight and obesity in Chinese adults.^[25]^

*Sarcopenic obesity:* study participants exhibiting both sarcopenia and obesity.

*Hypertension:* hypertension was defined by a systolic blood pressure ≥ 140 mm Hg, a diastolic blood pressure ≥ 90 mm Hg, or a past hypertension diagnosis with corresponding antihypertensive treatment.

*Diabetes:* individuals with a fasting blood glucose (FBG) level ≥ 7.0 mmol/L or a diagnosis of type 2 diabetes and a history of prior antidiabetic treatment.

*Smoking and drinking:* in accordance with standard World Health Organization definitions, smoking was defined as continuous smoking or smoking for 6 + months, while drinking was defined as alcohol consumption at least once per week on average with an average alcohol intake of 8 g/week.

### 2.4. Methylation analyses

Leukocyte genomic DNA was extracted with the PAXgene Blood DNA kit, and bisulfite sequencing was then used to assess *TWEAK/Fn14* methylation status. Briefly, following the bisulfite treatment of genomic DNA, polymerase chain reaction amplification was performed using primers specific for both ends of target CpG islands. Following purification of the amplified products, the target products were cloned and inserted into TA, with positive clones then being selected for sequencing. The BIQ-analyzer software was then utilized as a means of comparing the measured and original sequences, enabling the identification and enumeration of methylated sites and the calculation of the degree of methylation.

### 2.5. ELISAs

The ELISA kits were used to detect the concentrations of IL-4, IL-6, IL-10, TNF-α, and TWEAK in the plasma of 120 subjects. These kits included: ELISA-IL-4 kit (ek104-96 with coefficient of variance (CV of 4.5–5.1%, recovery = 0.94), Hangzhou Lianke Biotechnology Co., Ltd., Hangzhou, China), ELISA-IL-6 kit (ek106/2-96 (CV of 2.5–5.0%, recovery = 1.06), Hangzhou Lianke Biotechnology Co., Ltd.), ELISA-TNF-α kit (ek182-96 (CV of 3.8–8.9%, recovery = 0.93), Hangzhou Lianke Biotechnology Co., Ltd.), and ELISA-TWEAK kit (ek1256-96 (CV of 3.7–6.6%, recovery = 1.05), Hangzhou Lianke Biotechnology Co., Ltd.). All analyses were performed according to the provided instructions. Finally, the data were analyzed using ELISACalc software. The calculation formula is: (OD_Sample_ - OD_Blank_)/(OD_Standard_ - OD_Blank_) × 100%.

### 2.6. Statistical analyses

All data analyses were conducted using SPSS 17.0 (SPSS Inc., Chicago, IL) and R software (version 4.3.3). Results are reported as mean ± standard deviation (mean ± SD). Student *t* tests were employed to compare methylation rates or inflammatory mediator levels between the 2 groups, chi-square tests were used for categorical variables, multivariate logistic regression analysis was used for adjusting confounders (age, sex, triglyceride levels, hypertension, and diabetes) known to be associated with obesity and its complications.^[26]^ After adjusting for these same confounders, partial correlation analysis was used to examine the relationships between TWEAK levels, TNF-α levels, and TWEAK/Fn14 methylation. Furthermore, the correlation analysis between methylation or inflammatory factors and sarcopenic obesity, calculating odds ratios (ORs) and their 95% confidence intervals (CIs). Differences were considered statistically significant when *P* < .05.

## 3. Results

### 3.1. Participant characteristics

Study participant clinical characteristics are summarized in Table 1. Significant differences in age, triglyceride levels, and the prevalence of diabetes and hypertension were noted when comparing the case and control groups (*P* = .040, *P* = .005, *P* = .034, *P* = .003). No significant differences between these groups were observed with respect to serum creatinine, serum urea nitrogen, total cholesterol, high-density lipoprotein-cholesterol, low-density lipoprotein-cholesterol, gender, smoking history, or drinking history (*P* > .05).

**Table 1:** Clinical characteristics of the study participants [n (%), ±s].

	Control group	Case group	*t/Z/*χ^*2*^	*P*
	(n = 60)	(n = 60)		
Age (year)	71.24 ± 4.51	69.51 ± 3.91	2.244	.027
Serum creatinine (µmol/L)	65.26 ± 17.26	63.44 ± 17.88	0.569	.571
Serum urea nitrogen (mmol/L)	5.81 (4.97, 6.87)	5.93 (4.72, 7.40)	0.334	.739
Total cholesterol (mmol/L)	4.33 (3.92, 5.07)	4.60 (3.95, 5.18)	1.292	.196
Triglycerides (mmol/L)	1.04 (0.74, 1.57)	1.28 (0.92, 1.92)	2.063	.039
HDL-c (mmol/L)	1.30 (1.08, 1.55)	1.15 (0.98, 1.54)	1.572	.116
LDL-c (mmol/L)	2.59 ± 0.98	2.76 ± 1.01	-0.971	.333
Gender (Male, %)	25 (41.67)	27 (45.00)	0.034	.853
Smoking (Yes, %)	9 (15.00)	8 (13.33)	0.069	.793
Drinking (Yes, %)	7 (11.67)	7 (11.67)	<0.001	1.000
Diabetes (Yes, %)	7 (11.67)	16 (26.67)	4.357	.037
Hypertension (Yes, %)	28 (46.67)	42 (70.00)	6.720	.010

HDL-c = high-density lipoprotein-cholesterol, LDL-c = low-density lipoprotein-cholesterol.

### 3.2. The association between sarcopenic obesity and TWEAK and Fn14 methylation status

The association between sarcopenic obesity and *TWEAK*/*Fn14* methylation in community-dwelling elderly was next evaluated (Tables 2 and 3). Relative to the case group, individuals in the control group exhibited higher levels of *TWEAK* CpG20, CpG30, and CpG38 methylation and *Fn14* CpG25 methylation (*P* = .043, *P* = .043, *P* = .040, and *P* = .025, respectively).

**Table 2:** Methylation of *TWEAK* in community-dwelling older adult of Xinjiang.

	Control group	Case group	*Z/t*	*P*
CpG1	0.00 (0.00, 0.00)	0.00 (0.00, 0.00)	-0.096	.923
CpG2	0.00 (0.00, 0.00)	0.00 (0.00, 0.00)	-1.013	.311
CpG3	0.00 (0.00, 2.50)	0.00 (0.00, 10.00)	-1.029	.303
CpG4	0.00 (0.00, 0.00)	0.00 (0.00, 0.00)	-0.072	.942
CpG5	0.00 (0.00, 0.00)	0.00 (0.00, 0.00)	-1.061	.289
CpG6	0.00 (0.00, 2.50)	0.00 (0.00, 10.00)	-1.112	.266
CpG7	0.00 (0.00, 0.00)	0.00 (0.00, 0.00)	-0.502	.616
CpG8	0.00 (0.00, 10.00)	0.00 (0.00, 0.00)	-1.919	.055
CpG9	0.00 (0.00, 0.00)	0.00 (0.00, 0.00)	0.000	1.000
CpG10	0.00 (0.00, 0.00)	0.00 (0.00, 0.00)	-0.550	.582
CpG11	0.00 (0.00, 2.50)	0.00 (0.00, 10.00)	-0.336	.737
CpG12	0.00 (0.00, 0.00)	0.00 (0.00, 0.00)	-1.398	.162
CpG13	0.00 (0.00, 2.50)	0.00 (0.00, 0.00)	-1.697	.090
CpG14	0.00 (0.00, 0.00)	0.00 (0.00, 0.00)	0.000	1.000
CpG15	0.00 (0.00, 0.00)	0.00 (0.00, 5.00)	-1.363	.173
CpG16	0.00 (0.00, 0.00)	0.00 (0.00, 0.00)	-1.398	.162
CpG17	0.00 (0.00, 0.00)	0.00 (0.00, 9.55)	-1.046	.295
CpG18	0.00 (0.00, 2.50)	0.00 (0.00, 0.00)	-1.716	.086
CpG19	0.00 (0.00, 0.00)	0.00 (0.00, 0.00)	-0.416	.678
CpG20	0.00 (0.00, 0.00)	0.00 (0.00, 0.00)	-2.025	.043*
CpG21	0.00 (0.00, 2.50)	0.00 (0.00, 0.00)	-0.808	.419
CpG22	0.00 (0.00, 10.00)	0.00 (0.00, 0.00)	-1.597	.110
CpG23	0.00 (0.00, 0.00)	0.00 (0.00, 0.00)	-0.459	.646
CpG24	0.00 (0.00, 0.00)	0.00 (0.00, 0.00)	-0.024	.981
CpG25	0.00 (0.00, 0.00)	0.00 (0.00, 0.00)	-0.977	.329
CpG26	0.00 (0.00, 0.00)	0.00 (0.00, 5.00)	-1.212	.226
CpG27	0.00 (0.00, 0.00)	0.00 (0.00, 0.00)	-1.733	.083
CpG28	0.00 (0.00, 0.00)	0.00 (0.00, 0.00)	-1.465	.143
CpG29	0.00 (0.00, 0.00)	0.00 (0.00, 0.00)	-1.013	.311
CpG30	0.00 (0.00, 0.00)	0.00 (0.00, 0.00)	-2.026	.043*
CpG31	0.00 (0.00, 0.00)	0.00 (0.00, 0.00)	-0.809	.418
CpG32	0.00 (0.00, 0.00)	0.00 (0.00, 0.00)	-0.990	.322
CpG33	0.00 (0.00, 0.00)	0.00 (0.00, 0.00)	-0.550	.582
CpG34	0.00 (0.00, 0.00)	0.00 (0.00, 0.00)	-0.096	.923
CpG35	0.00 (0.00, 0.00)	0.00 (0.00, 10.00)	-1.189	.234
CpG36	0.00 (0.00, 0.00)	0.00 (0.00, 0.00)	-0.378	.705
CpG37	0.00 (0.00, 0.00)	0.00 (0.00, 0.00)	-0.480	.631
CpG38	50.00 (37.50, 62.50)	55.56 (50.00,71.36)	-1.887	.059

TWEAK = tumour necrosis factor-like weak inducer of apoptosis.

* The methylation of *TWEAK* in control group was significantly higher than that in case group.

**Table 3:** Methylation of *Fn14* in community-dwelling older adult of Xinjiang.

	Control group	Case group	*Z*	*P*
CpG1	90.00 (72.05, 100.00)	81.82 (70.00, 90.45)	-1.104	.270
CpG2	80.00 (70.00, 90.00)	81.82 (70.00, 90.00)	-0.012	.990
CpG3	95.00 (87.50, 100.00)	100.00 (90.00, 100.00)	-1.094	.274
CpG4	90.00 (80.00, 100.00)	90.00 (80.91, 95.45)	-0.541	.589
CpG5	95.00 (80.00, 100.00)	90.91 (80.91, 100.00)	-0.052	.959
CpG6	80.00 (62.73, 90.00)	80.00 (70.00, 90.00)	-0.148	.882
CpG7	52.27 (44.09, 70.00)	50.00 (45.00, 80.00)	-0.370	.711
CpG8	30.00 (20.00, 50.00)	20.00 (20.00, 40.00)	-1.291	.197
CpG9	20.00 (16.14, 30.00)	20.00 (10.00, 30.00)	-0.545	.586
CpG10	19.09 (0.00, 22.50)	10.00 (9.09, 20.00)	-0.222	.824
CpG11	0.00 (0.00, 2.27)	0.00 (0.00, 9.55)	-0.286	.775
CpG12	10.00 (10.00, 22.50)	10.00 (0.00, 20.00)	-1.281	.200
CpG13	10.00 (0.00, 10.00)	10.00 (0.00, 20.00)	-0.894	.371
CpG14	0.00 (0.00, 9.32)	0.00 (0.00, 10.00)	-1.037	.300
CpG15	10.00 (10.00, 20.00)	10.00 (0.00, 20.00)	-0.809	.419
CpG16	28.64 (0.00,40.00)	20.00 (9.09, 30.00)	-0.712	.476
CpG17	10.00 (6.82, 30.00)	9.09 (0.00, 25.00)	-0.992	.321
CpG18	20.00 (10.00, 32.50)	27.27 (10.00, 35.00)	-0.430	.667
CpG19	10.00 (0.00, 10.00)	0.00 (0.00, 10.00)	-0.894	.371
CpG20	14.09 (10.00, 20.00)	10.00 (0.00, 20.00)	-0.896	.370
CpG21	0.00 (0.00, 0.00)	0.00 (0.00, 10.00)	-0.695	.487
CpG22	0.00 (0.00, 10.00)	0.00 (0.00, 0.00)	-1.485	.137
CpG23	0.00 (0.00, 2.5)	0.00 (0.00, 9.55)	-0.350	.727
CpG24	0.00 (0.00, 2.5)	0.00 (0.00, 0.00)	-0.361	.718
CpG25	0.00 (0.00, 10.00)	0.00 (0.00, 0.00)	-2.243	.025*
CpG26	0.00 (0.00, 10.00)	0.00 (0.00, 0.00)	-0.990	.322
CpG27	0.00 (0.00, 2.273)	0.00 (0.00, 5.00)	-0.083	.934
CpG28	0.00 (0.00, 2.273)	0.00 (0.00, 4.55)	-0.099	.922
CpG29	0.00 (0.00, 2.50)	0.00 (0.00, 0.00)	-1.286	.198
CpG30	0.00 (0.00, 0.00)	0.00 (0.00, 0.00)	-0.246	.806

Fn14 = fibroblast growth factor-inducible 14.

* The methylation of *Fn14* in control group was significantly higher than that in case group.

### 3.3. Associations between sarcopenic obesity and inflammatory factors

**Figure 1.**
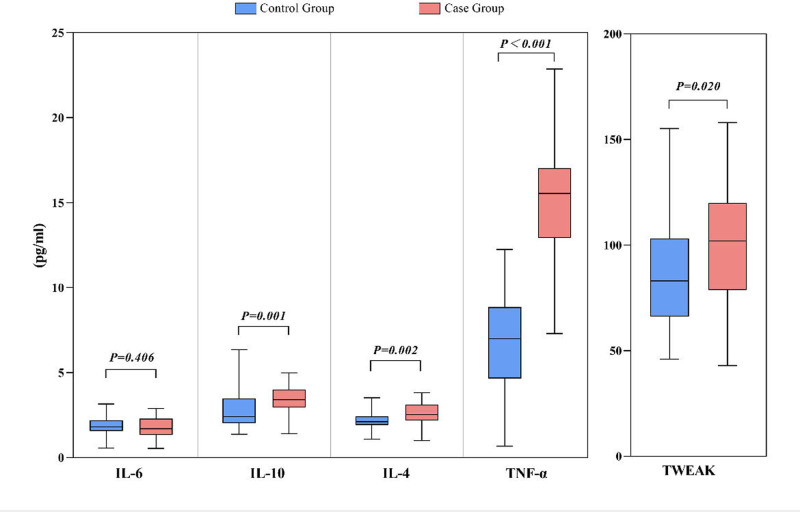
The cytokine difference analysis between case and control groups.

Control subjects exhibited significantly lower plasma TWEAK, TNF-α, IL-10, and IL-4 levels relative to individuals in the case group (*P* = .020, *P* < .001, *P* = .001, *P* = .002, respectively) (Fig. 1). Following adjustment for age, sex, triglyceride levels, diabetes, and hypertension, a covariate variance analysis revealed significant differences between these groups with respect to plasma levels of TWEAK, TNF-α, IL-10, and IL-4 (*P*_TWEAK_ = .017, *P*_TNF-α_ < .001, *P*_IL-10_ = .010, *P*_IL-4_ = .016, Table 4).

**Table 4:** Plasma IL-6, IL-10, IL-4, TNF-α, and TWEAK between control and case group.

Groups	IL-6 (pg/mL)	IL-10 (pg/mL)	IL-4 (pg/mL)	TNF-α (pg/mL)	TWEAK (pg/mL)
*t* test					
Control group	1.87 ± 0.571	2.8 ± 1.095	2.23 ± 0.548	6.63 ± 2.926	87.67 ± 27.333
Case group	1.78 ± 0.599	3.43 ± 0.87	2.58 ± 0.615	15.22 ± 3.292	99.35 ± 26.923
*t*	0.834	3.502	3.240	15.107	2.359
*P*	0.406	0.001	0.002	﹤0.001	0.020
Covariate variance analysis					
Control group*	1.891	2.860	2.274	6.778	86.617
Case group*	1.746	3.377	2.553	15.034	100.113
*F*	1.490	6.776	5.950	175.372	5.852
*P*	0.225	0.010	0.016	﹤0.001	0.017

IL-10 = interleukin 10, IL-4 = interleukin 4, IL-6 = interleukin 6, TNF-α = tumour necrosis factor-alpha, TWEAK = tumour necrosis factor-like weak inducer of apoptosis.

* Mean of IL-6, IL-10, IL-4, TNF-α, and TWEAK after adjusted for age, gender, triglycerides, diabetes, and hypertension.

### 3.4. Correlations between inflammatory mediators and TWEAK/Fn14 methylation

Following adjustment for age, sex, triglyceride levels, diabetes, and hypertension, partial correlation analysis revealed that plasma TWEAK levels were correlated with TNF-α levels (*R* = 0.211, *P* = .029) and *Fn14* CpG25 methylation (*R* = 0.431, *P* = .008), whereas these levels were not correlated with plasma IL-4, IL-6, or IL-10 (*P* = .751, *P* = .079, *P* = .704; Table 5).

**Table 5:** Partial correlation analysis of TWEAK levels in plasma.

	*r*	*P*
TNF-α levels	0.211	.029
IL-4 levels	0.030	.751
IL-6 levels	-0.175	.079
IL-10 levels	-0.037	.704
Fn14 CpG25 methylation	0.431	.008

Fn14 = fibroblast growth factor-inducible 14, IL-10 = interleukin 10, IL-4 = interleukin 4, IL-6 = interleukin 6, TNF-α = tumour necrosis factor-alpha, TWEAK = tumour necrosis factor-like weak inducer of apoptosis.

### 3.5. Multivariate analyses of the relationships between plasma TWEAK and TNF-α levels and the incidence of sarcopenic obesity

Following adjustment for relevant confounding factors (age, sex, triglycerides, diabetes, and hypertension), multivariate logistic regression analyses indicated that higher plasma levels of TWEAK and TNF-α were significantly associated with the incidence of sarcopenic obesity (OR = 1.024, 95% CI = 1.006–1.042; OR = 2.537, 95% CI = 1.697–3.793; Table 6).

**Table 6:** Odds ratios and 95% confidence intervals for TWEAK, TNF-α associated with sarcopenic obesity.

	B	SE	Wald χ² value	*P*	OR	95% CI	
						Lower	Upper
Model 1							
TWEAK	0.024	0.009	6.838	.009	1.024	1.006	1.042
Gender (Female)	-0.595	0.476	1.567	.211	0.551	0.217	1.400
Age (yr)	-0.223	0.064	12.084	.001	0.800	0.706	0.907
Diabetes (yes)	1.086	0.624	3.031	.082	2.961	0.872	10.052
Hypertension (yes)	1.153	0.490	5.541	.019	3.169	1.213	8.277
Triglycerides (mmol/L)	1.057	0.372	8.051	.005	2.877	1.387	5.969
Model 2							
TNF-α	0.931	0.205	20.599	﹤.001	2.537	1.697	3.793
Gender (Female)	0.247	0.885	0.078	.780	1.280	0.226	7.245
Age (yr)	0.046	0.107	0.182	.670	1.047	0.849	1.290
Diabetes (yes)	0.351	0.900	0.152	.696	1.421	0.243	8.292
Hypertension (yes)	0.728	0.823	0.783	.376	2.071	0.413	10.387
Triglycerides (mmol/L)	0.638	0.604	1.116	.291	1.893	0.579	6.184

95% CI = 95% confidence intervals, OR = odds ratios, SE = standard error, TNF-α = tumour necrosis factor-alpha, TWEAK = tumour necrosis factor-like weak inducer of apoptosis.

## 4. Discussion

This study demonstrated that lower levels of *TWEAK/Fn14* methylation are significantly associated with the incidence of sarcopenic obesity among the Xinjiang elderly. In addition, higher plasma concentrations of TNF-α and TWEAK were found to be significantly associated with sarcopenic obesity.

The aging process is associated with many changes in body composition, human muscle mass peaks at the age of 40 and thereafter declines, while average fat mass rises with age. Muscle loss accelerates after 70 years of age, while age-related intramuscular fat infiltration can become increasingly prevalent. Obesity can similarly contribute to the adipose tissue infiltration of the liver, skeletal muscle, and other organs. This extensive lipid deposition within muscle cells can spur inflammatory activity and lipotoxicity, inducing the dedifferentiation of mesenchymal progenitor cells expressing adipose tissue-related genes. These processes compromise normal muscle regeneration and contribute to reduced mitochondrial counts, reduced lipolysis, and impaired fatty acid oxidation, thereby promoting the emergence of insulin resistance and the disruption of muscle function.^[27]^ In our preliminary epidemiological analysis, we also found a certain correlation between the obesity index in the Xinjiang region and age.

Skeletal muscle accounts for 30% to 40% of the body weight of healthy adults, comprising the largest functional tissue type in the body. Skeletal muscle utilizes approximately 80% of available glucose in the body, and skeletal muscle disorders can thus contribute to abnormal glucose homeostasis and insulin sensitivity. In order to maintain normal metabolic activity, it is thus essential that physiological skeletal muscle mass and function be preserved.^[28,29]^ Reductions in skeletal muscle mass or the infiltration of muscle tissue by adipose tissue can contribute to reductions in strength, insulin resistance, and dyskinesia. In obese individuals, insulin resistance can also promote muscle catabolism, which is independently associated with the risk of declining muscle strength. The abnormal levels of triglycerides, as well as diabetes and hypertension, displayed in the case group of this study also confirm this point. Therefore, we speculate that sarcopenia and obesity in elderly patients are inseparable in their stages of occurrence.

TWEAK and its receptor Fn14 comprise a recently established signal transduction pathway that is closely associated with the incidence of both acute and chronic forms of muscle atrophy. This pathway shapes the synthesis and degradation of proteins through a range of mechanisms, ultimately interfering with muscle synthesis and increasing catabolic activity, thereby contributing to consequent reductions in the mass and function of skeletal muscle.^[30–32]^ Some reports have also found that Fn14 expression levels in the skeletal muscle rise with age, potentially as a consequence of DNA methylation or other epigenetic changes that result in enhanced TWEAK-Fn14 signaling activity and related alterations in skeletal muscle metabolism.^[33]^ The study worthy of note has confirmed that this perspective occurs in the bodies of elderly patients with sarcopenic obesity in the Xinjiang community. Specific changes include a decrease in TWEAK/Fn14 methylation and an increase in the circulating concentrations of TWEAK and TNF-α. Furthermore, our research also provides evidence that the plasma levels of TWEAK are closely related to TNF-α concentrations and the methylation status of Fn14. The underlying mechanism of hypomethylation of TWEAK/Fn14 promoting the upregulation of TWEAK/Fn14 and facilitating the activation of inflammatory signaling pathways for sarcopenic obesity progression needs to be further verified.

Key overlapping inflammatory pathways can contribute to both fat accumulation and muscle loss. The aging process is associated with higher concentrations of TNF-α and other proinflammatory cytokines, while obesity can similarly promote chronic low-grade inflammation in addition to activating T cells, mast cells, and macrophages. These inflammatory factors can, in turn, promote insulin resistance that ultimately accelerates the degradation of protein by muscle tissue and contributes to apoptotic death, with the net effect of inducing adipose tissue formation and muscle tissue degradation.^[7,21]^ This explains our research on detecting the changes in the expression of inflammatory factors in the plasma of patients with sarcopenic obesity. TWEAK is a tumor necrosis factor superfamily protein that, together with Fn14, has been reported to be upregulated in subcutaneous and visceral adipose tissue samples from obese individuals.^[34]^ Bennett et al studied TWEAK-transgenic mice expressing 3-fold higher TWEAK protein levels and found that these mice exhibited pronounced weight gain, insulin resistance, metabolic disorders, and significantly reduced exercise endurance.^[35]^ We also observed this abnormal expression in the bodies of patients with sarcopenic obesity.

TWEAK-Fn14 axis is a major upstream target of nuclear factor kappa-B (NF-κB) signaling axis.^[36]^ It is central to the incidence and progression of sarcopenia.^[37]^ By activating NF-κB signaling, TWEAK can trigger a series of reactions.^[38]^ TNF-α is a major inflammatory factor produced in response to this NF-κB activation that has been mechanistically linked to sarcopenia development. Both humans and animals exhibiting severe skeletal muscle loss present with elevated serum concentrations of TNF-α.^[39,40]^ Early in vitro TNF-α treatment has been shown to enhance the methylation of muscle transcriptional regulator genes within muscle cells through a form of so-called “memory.”^[41]^ Here, sarcopenic obesity was found to be associated with significantly elevated plasma TNF-α and TWEAK concentrations, and the levels of these 2 factors were also strongly correlated with one another. When produced in response to appropriate signals, insulin can promote the storage of the majority of circulating glucose in the form of glycogen for use by the skeletal muscle. The skeletal muscle uses a large proportion of available glucose. Given the high total skeletal muscle mass and its high degree of insulin responsivity, reductions in muscle mass and/or function can thus partially compromise normal blood glucose maintenance while reducing the resting metabolic rate and total energy consumption, thereby promoting the accumulation of fat and driving more rapid weight gain.^[42,43]^ Given the present results, we demonstrated the crucial association of *TWEAK/Fn14* hypomethylation and the levels of TWEAK and TNF-α affecting sarcopenic obesity through their impacts on skeletal muscle function and quality.

These analyses have certain limitations. Firstly, due to the cross-sectional design, causality cannot be established between TWEAK/Fn14 hypomethylation and sarcopenic obesity. Secondly, the study focuses on a small, region-specific sample from Xinjiang, limiting its generalizability. Additionally, there is a lack of mechanistic evidence proving the link between hypomethylation and sarcopenic obesity. Confounding factors such as physical activity, nutrition, comorbidities, and medications were not comprehensively addressed, and the potential for reverse causation was not explored. At present study, our research preliminarily elaborates on the hypomethylation of TWEAK/Fn14 and the changes in plasma-related factors affecting sarcopenic obesity progression. In future research, we will collaborate with multiple centers to collect Sarcopenic obesity cases from a wider range of regions, age groups, and ethnicities. Additionally, we will incorporate more diagnostic methods in our future studies to gain a clearer understanding of the etiology of sarcopenic obesity. Our research will also not be limited to clinical analysis.

Despite these limitations, the study has several strengths. The Xinjiang population’s stability in lifestyle and environment provides valuable insights into genetic factors contributing to sarcopenia. Furthermore, the random selection of participants from the elderly community population ensures credible data collection. In future research, we will collaborate with multiple centers to collect sarcopenic obesity cases from a wider range of regions, age groups, and ethnicities. We will also incorporate more diagnostic methods to gain a clearer understanding of the etiology of sarcopenic obesity, ensuring a comprehensive analysis that addresses these limitations.

## 5. Conclusion

In summary, our study revealed that *TWEAK/Fn14* hypomethylation and increases in plasma TWEAK and TNF-α concentrations are associated with the progression of sarcopenic obesity, providing an important foundation for future research efforts.

## Acknowledgments

We would like to express our sincere gratitude to the director of the Public Health Bureau for their continuous support of our population survey in the Mulei and Luofu areas. We thank all the staff of the Second Department of Cadre Health Care Centre of People’s Hospital of Xinjiang Uygur Autonomous Region for support with the medical examination and demographic data collection.

## Author contributions

**Conceptualization:** Lingling Ma, Hongmei Wang.

**Data curation:** Zhuoya Maimaitiwusiman, Saiyare Xuekelati, Hongmei Wang.

**Formal analysis:** Buluhan Halan, Jinling Liu, Hongmei Wang.

**Visualization:** Buluhan Halan, Jinling Liu.

**Writing – original draft:** Lingling Ma.

**Writing – review & editing:** Lingling Ma, Zhuoya Maimaitiwusiman, Saiyare Xuekelati, Buluhan Halan, Jinling Liu, Hongmei Wang.
